# Supplementary material for: Effector prediction and characterization in the oomycete pathogen Bremia lactucae reveal host-recognized WY domain proteins that lack the canonical RXLR motif
Source: PLoS Pathog. 2020 Oct 26;16(10):e1009012. doi: 10.1371/journal.ppat.1009012 (PMC7644090; doi:10.1371/journal.ppat.1009012)
Supplement: S1 Table — Sequences that occur in more than one protein are highlighted in red. (DOCX) [file ppat.1009012.s001.docx]

| *Starting with:* | **Glycine** | **Histidine** | **Lysine** | **Glutamine** | **Arginine** |
| --- | --- | --- | --- | --- | --- |
|  | GCLR | HAVN | KALN | QAIN | RAVN |
|  | GDMN | HFR | KDFK | QEIN | RFK |
|  | GELK | HIK | KDLN | QELR | RFN |
|  | GHLK | HILN | KFK | QFN | RFR |
|  | GKMK | HIR | KFMN | QGVR | RFVK |
|  | GLFK | HLK | KIK | QIIK | RHLN |
|  | GLK | HLN | KIMR | QLN | RIR |
|  | GLLN | HLR | KIR | QLR | RIYR |
|  | GLN | HMN | KIVK | QLVK | RIYR |
|  | GVK | HSFK | KKLK | QLVN | RLK |
|  |  | HSVN | KLIK | QSIR | RLR |
|  |  | HYVK | KLK | QTFK | RMLK |
|  |  |  | KLN | QTFN | RMN |
|  |  |  | KLR | QVVR | RPLK |
|  |  |  | KMFK | QVYK | RPVN |
|  |  |  | KRLK | QYLK | RQFN |
|  |  |  | KSFK |  | RRLN |
|  |  |  | KSVK |  | RSIR |
|  |  |  | KSVN |  | RSLK |
|  |  |  | KVFK |  | RTIK |
|  |  |  | KVK |  | RVFK |
|  |  |  | KVLN |  | RVK |
|  |  |  | KVN |  | RVR |
|  |  |  | KVR |  | RYLK |
|  |  |  | KYR |  | RYNK |
