# Supplementary material for: Effector prediction and characterization in the oomycete pathogen Bremia lactucae reveal host-recognized WY domain proteins that lack the canonical RXLR motif
Source: PLoS Pathog. 2020 Oct 26;16(10):e1009012. doi: 10.1371/journal.ppat.1009012 (PMC7644090; doi:10.1371/journal.ppat.1009012)
Supplement: S2 Table — (DOCX) [file ppat.1009012.s002.docx]

| **Species** | **RXLR+EER (with WY)** | **RXLR (no EER, with WY)** | **EER (no RXLR, with WY)** | **No RXLR or EER (with WY)** | **RXLR-EER (no WY)** |
| --- | --- | --- | --- | --- | --- |
| *B. lactucae* | 8 | 3 | 20 | 8 | 75 |
| *P. viticola* INRA-PV221 | 6 | 18 | 246 | 37 | 120 |
| *P. viticola* JL-7-2 | 7 | 6 | 166 | 30 | 98 |
| *P. halstedii* | 0 | 2 | 25 | 8 | 57 |
| *P. tabacina* | 15 | 1 | 16 | 2 | 52 |
| *P. effusa* | 30 | 13 | 15 | 6 | 67 |
| *H. arabidopsidis* | 5 | 1 | 27 | 11 | 81 |
| *P. cubensis* | 21 | 4 | 20 | 4 | 103 |
| *P. parasitica* | 96 | 16 | 23 | 8 | 222 |
| *P. infestans* | 123 | 21 | 35 | 11 | 310 |
| *P. capsici* | 52 | 8 | 12 | 4 | 165 |
| *P. cinnamomi* | 73 | 10 | 11 | 2 | 156 |
| *P. ramorum* | 107 | 5 | 16 | 0 | 147 |
| *P. sojae* | 81 | 18 | 12 | 2 | 240 |
