# Supplementary figures and images for: Effector prediction and characterization in the oomycete pathogen Bremia lactucae reveal host-recognized WY domain proteins that lack the canonical RXLR motif
Source: PLoS Pathog. 2020 Oct 26;16(10):e1009012. doi: 10.1371/journal.ppat.1009012 (PMC7644090; doi:10.1371/journal.ppat.1009012)

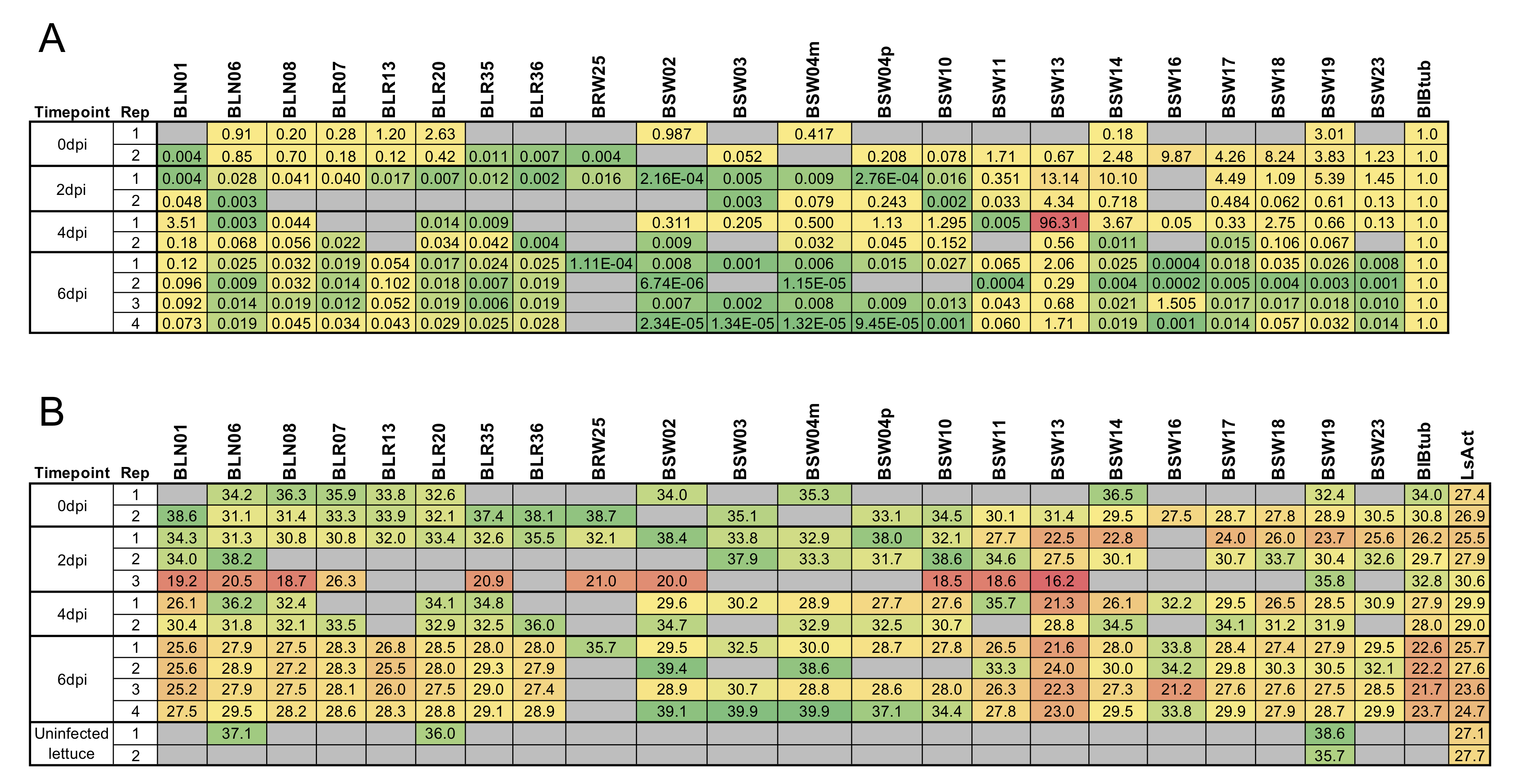

Supplement: S1 Fig — (A) Normalized expression levels for each effector transcript at 0, 2, 4, and 6 days post infection (dpi) and uninfected lettuce as a control. B. lactucae B-tubulin (BlBtub) was used as a housekeeping gene to quantify total amount of B. lactucae RNA on infected seedlings and L. sativa actin (LsAct) was used to quantify the amount of lettuce RNA. Transcript levels normalized to B-tubulin using the 2ΔCt method (2(Ct effector–Ct b-tubulin))[98]. (B) Raw Ct values for each effector transcript at 0, 2, 4, and 6 days post infection (dpi) and uninfected lettuce as a control. B. lactucae B-tubulin (BlBtub) was used as a housekeeping gene to quantify total amount of B. lactucae RNA on infected seedlings and L. sativa actin (LsAct) was used to quantify the amount of lettuce RNA. (TIFF) [file ppat.1009012.s003.tiff]

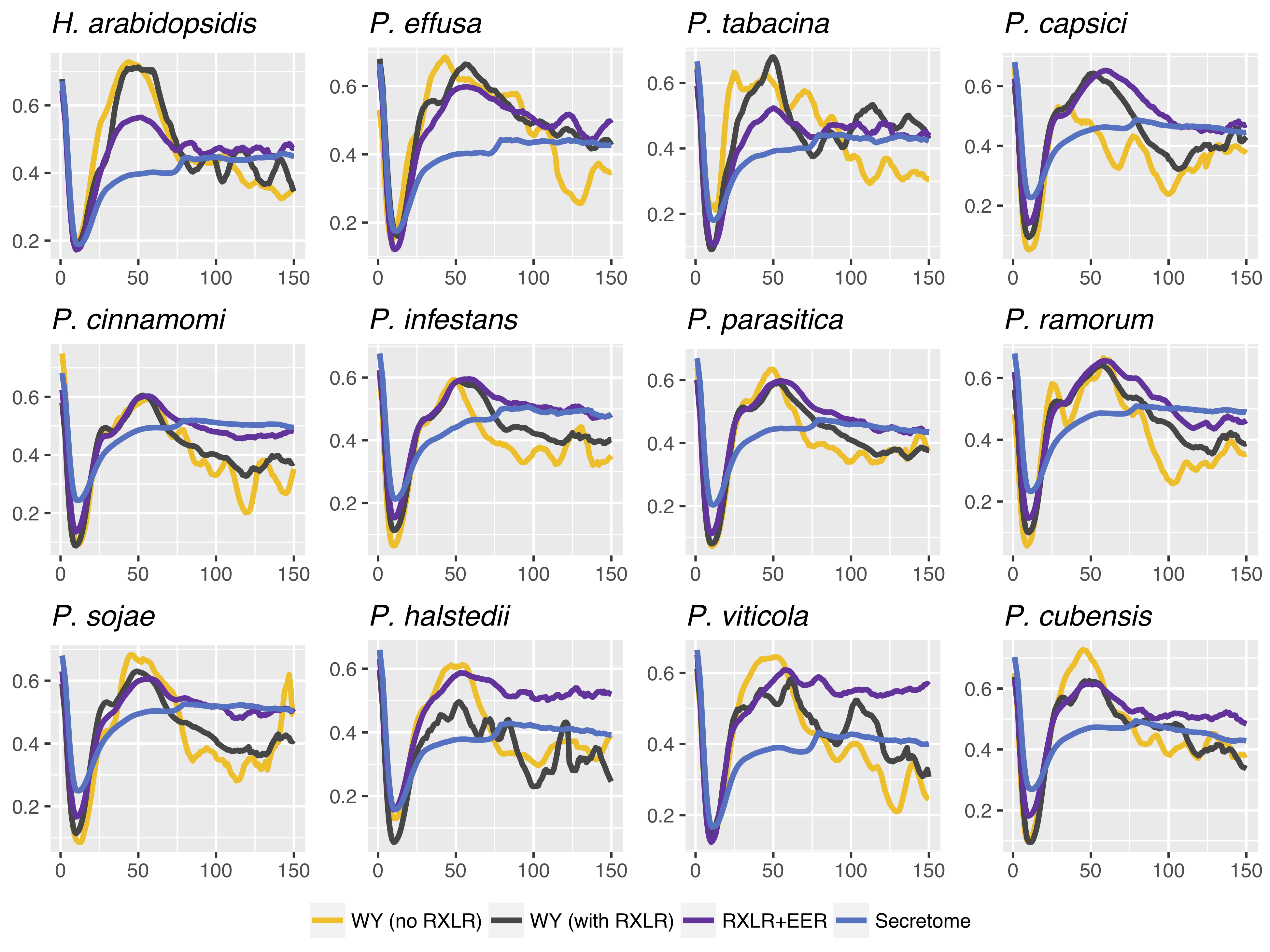

Supplement: S2 Fig — The x-axis shows the amino acid position; the y-axis shows the intrinsic disorder (VSL2 PONDR) score. Proteins were categorized as WY with no RXLR (yellow), WYs with RXLR (black), RXLR+EER (with or without WY, purple), and the total predicted secretome (blue) using the same color scheme as Fig 2. RXLR and EER motifs were as described in Figs 2 and 3. Average positional disorder scores are shown for each class of proteins. (TIFF) [file ppat.1009012.s004.tiff]

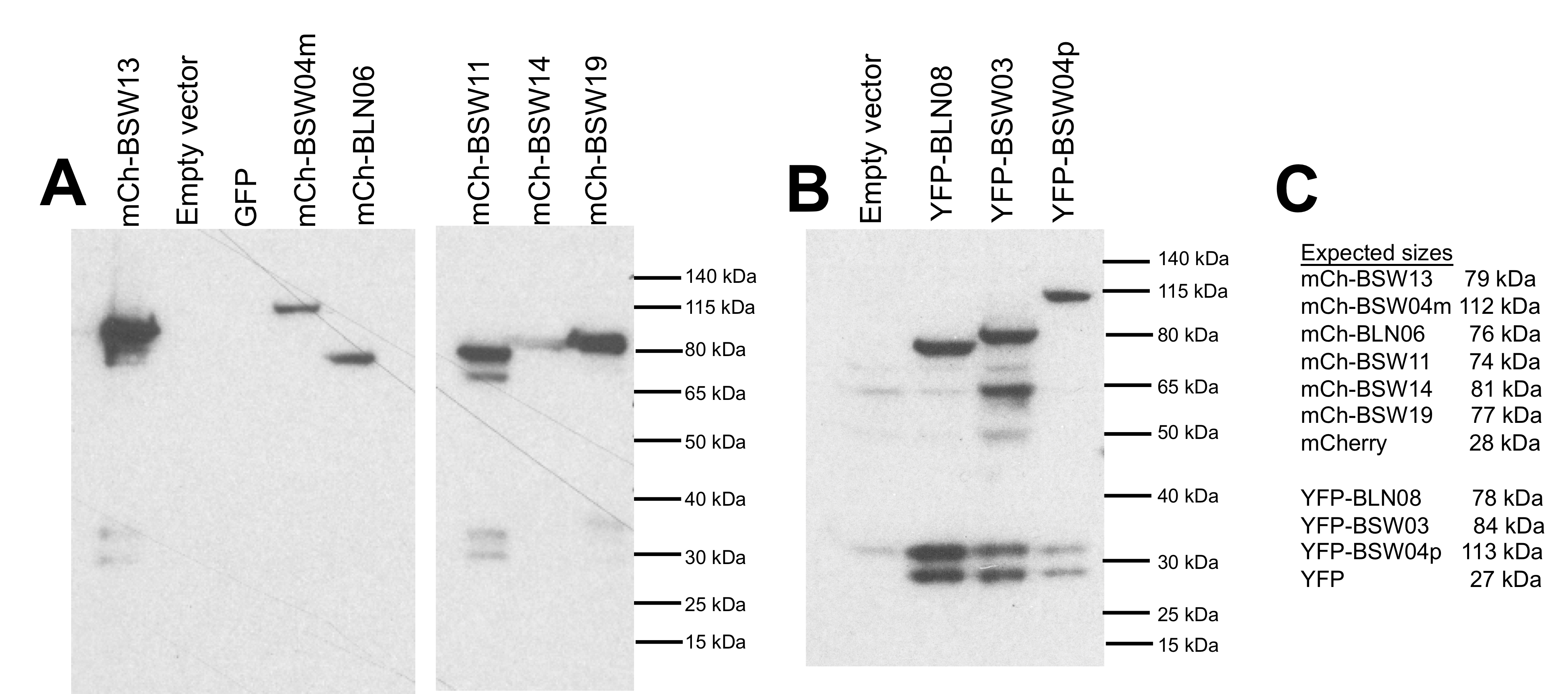

Supplement: S3 Fig — (A) Anti-mCherry (mCh) Western blot. (B) Anti-YFP Western blot. (C) Expected sizes for each of the fusion proteins. (TIFF) [file ppat.1009012.s005.tiff]

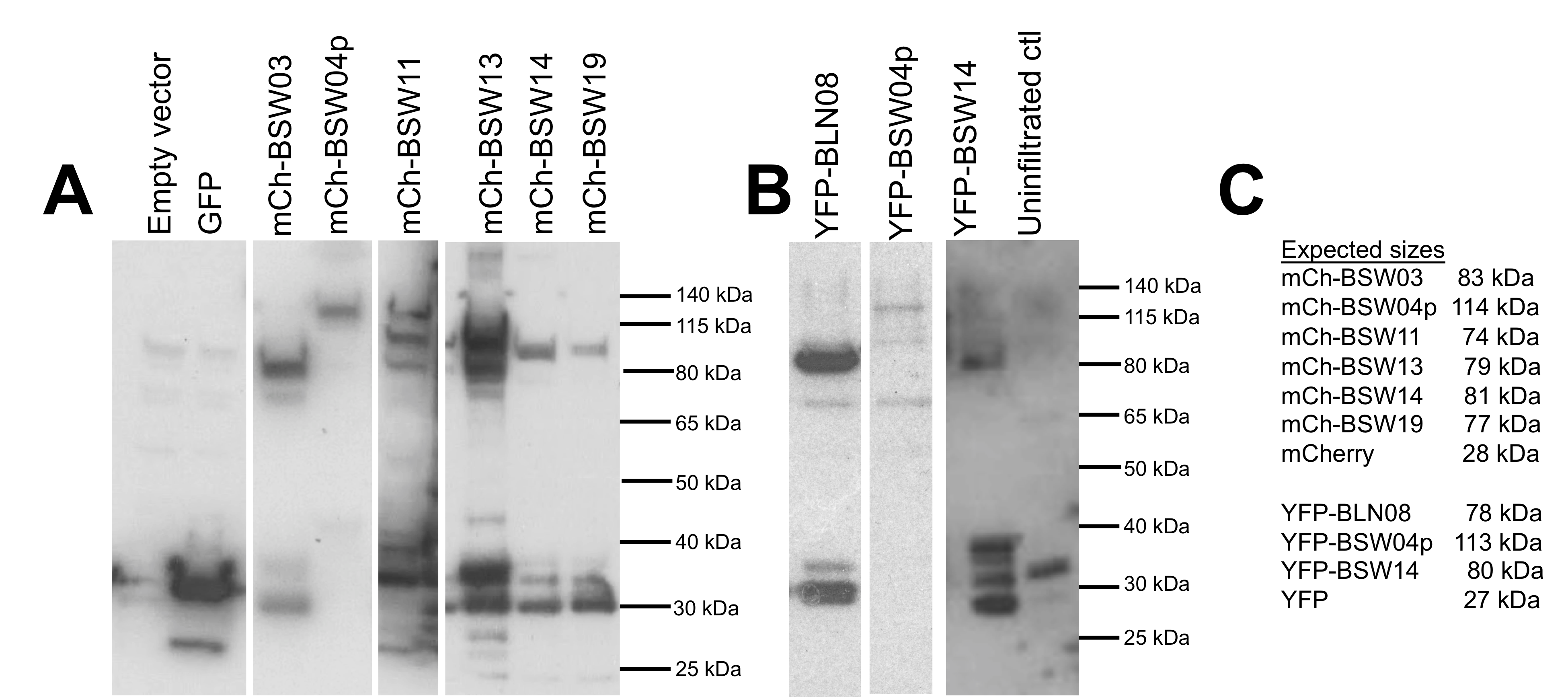

Supplement: S4 Fig — (A) Anti-mCherry (mCh) Western blot. (B) Anti-YFP Western blot. (C) Expected sizes for each of the fusion proteins. (TIFF) [file ppat.1009012.s006.tiff]

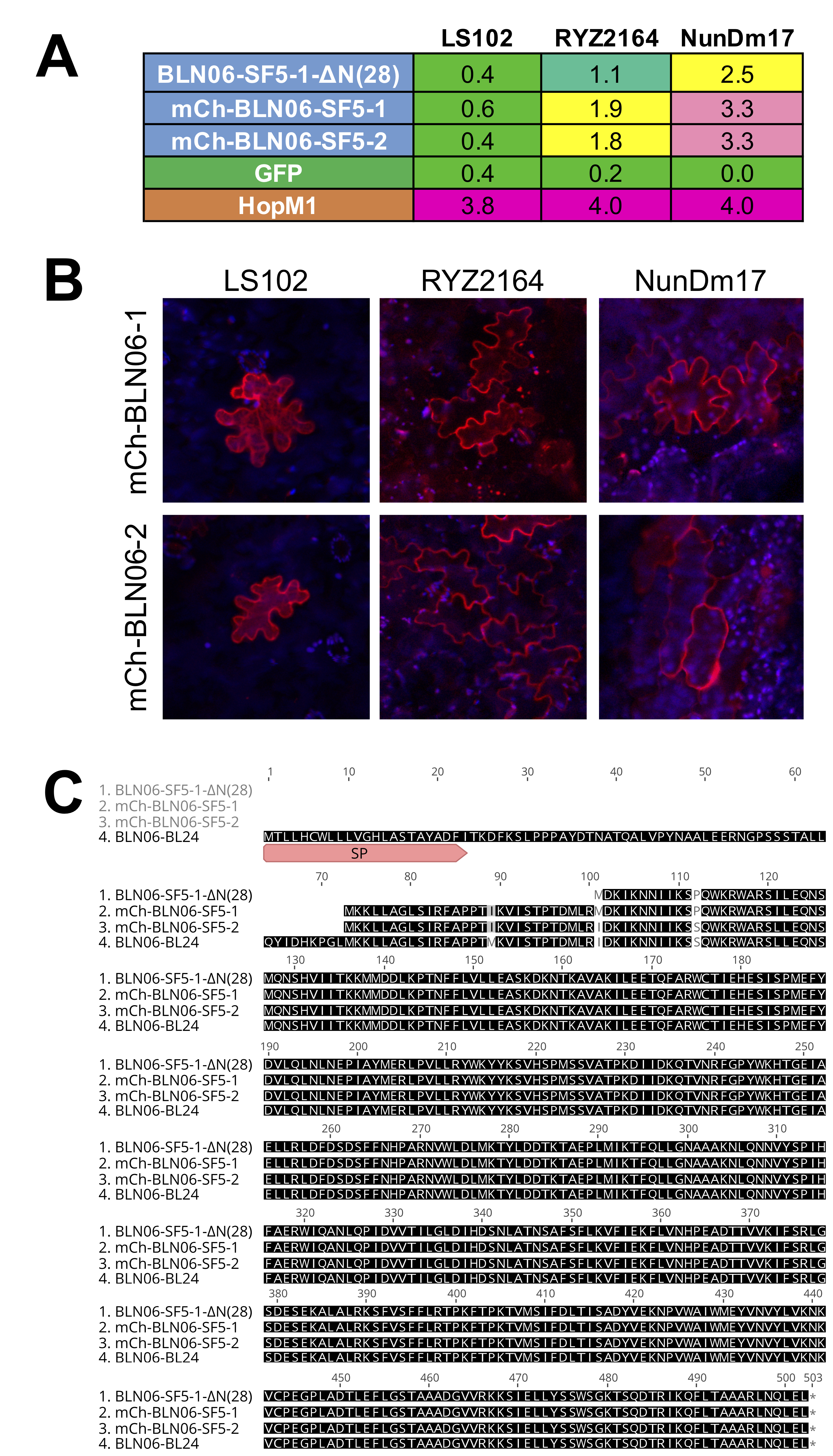

Supplement: S5 Fig — (A) Agroinfiltration scores from various isoforms of BLN06 from B. lactucae isolate SF5. Scores as in Fig 7. One leaf per plant was infiltrated and scores were averaged across 10 replicates. All leaves were confirmed to be expressing mCherry (mCh)-BLN06 by confocal microscopy. (B) Example confocal microscopy of N-terminal mCherry-tagged BLN06 in LS102, RYZ2164, and NunDm17 lettuce genotypes. (C) Sequence comparison between BLN06 isoforms from isolates SF5 and BL24 [45]. BLN06 was initially cloned from SF5 with the first 28 amino acids removed in case there was a cryptic signal peptide. For the mCherry-BLN06 fusions, these 28 amino acids were included in the protein sequence. Both alleles of BLN06 were cloned from SF5 (BLN06-SF5-1 and BLN06-SF5-2). (TIFF) [file ppat.1009012.s007.tiff]

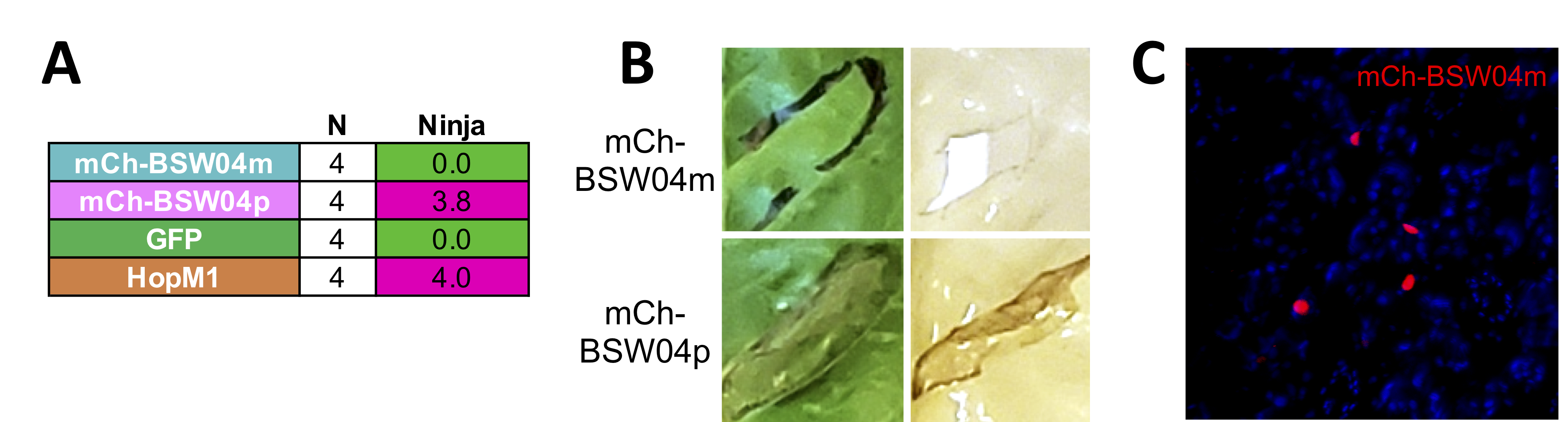

Supplement: S6 Fig — (A) Agroinfiltration scores from L. sativa cv Ninja three days post infiltration. GFP is used as a negative control, HopM1 is used as a positive control for necrosis. (B) Representative photo of infiltrated Ninja leaves before clearing (left) and after clearing (right) with ethanol to visualize necrosis (brown areas). (C) Example confocal microscopy result of mCherry-BSW04m (red) transiently expressed in Ninja. All four leaves showed similar expression and nuclear localization. Chlorophyll autofluorescence is shown in blue. The fusion protein is intact as measured by Western blot (S3 Fig). (TIFF) [file ppat.1009012.s008.tiff]
