# Supplementary material for: Effector prediction and characterization in the oomycete pathogen Bremia lactucae reveal host-recognized WY domain proteins that lack the canonical RXLR motif
Source: PLoS Pathog. 2020 Oct 26;16(10):e1009012. doi: 10.1371/journal.ppat.1009012 (PMC7644090; doi:10.1371/journal.ppat.1009012)
Supplement: Attachment — Submitted filename: Response-to-reviewers-20200920.docx [file ppat.1009012.s013.docx]

**Response to Reviewers**

**Part I - Summary**

**“Considering novelty, since the first submission of the manuscript there has been a publication reporting the identification of WY-domain proteins lacking RXLR motifs in a different downy mildew species (Combier et al, PLoS ONE, 14(7): e0220184). This paper should be cited, at least in the introduction.”**

Thanks for pointing this out. This has been added to Introduction (line 141).

**“Reviewer #3: The authors present bioinformatic prediction of candidate WY-containing effectors. It is disappointing that they have removed data indicating whether the effector candidates are up regulated during infection as this is a key criterion giving confidence to the assertion that they are indeed effectors.”**

Since *B. lactucae* is an obligate biotroph, all samples have to be collected during infection (besides those consisting of only ungerminated spores); therefore it is not possible to see which genes are upregulated during infection. It is possible to test whether genes are expressed earlier or later during infection; however, our number of replicates (3) was not sufficient to demonstrate statistical significance of the temporal trends observed in the removed RNA-seq experiment or in the new q-RT-PCR experiment. The fact that they are all expressed during infection is consistent with their potential role as effectors, which are the data that we have retained.

**“The work on localisation and PTI suppression remains preliminary and unconvincing.”**

Localization studies are by their nature descriptive and just part of the biology of an effector. The fact that we observe different patterns are indicative of different cellular localizations, subject to the same caveats of any localization study with fusions to fluorescent reporter proteins. We have added Western blots to demonstrate the presence of full-length fusion protein as requested.

We generated fusion proteins as requested previously to demonstrate expression in the PTI suppression assay. For technical reasons, fusion proteins were not obtained for all effectors. We found that those that were obtained had modified PTI suppression activity; this is not unexpected because the reporter protein can alter stability and/or function. We do not claim that the ones for which we did not observe PTI suppression might not also have this activity (for example, in the scenario that they were not expressed). The important point is that we consistently observed PTI suppression with three untagged WY effectors (along with 2 tagged effectors), which we believe to be true activities that are supported by statistical analysis of our data.

**“The triggering of cell death does not mean that an effector has been ‘recognised’ by a host resistance. It could also be because overexpression of the effector has become toxic to the plant cell. This can occur, for example, when excess effector could have off-target effects, or could prevent basic host cell functions, such as normal membrane integrity or fusions. Interestingly, overexpression of BSW14, for example, causes cell death in all saligna genotypes.”**

We agree. Consequently, we have changed the language throughout results; the text now says “triggers cell death” rather than “is recognized.” In the Discussion section (lines 462 to 568), we have added this a caveat. However, the fact that necrosis only occurs in specific lettuce genotypes suggests a specific interaction with R genes rather than a general toxicity of the effectors. It is not uncommon for a non-host species to consistently recognize an effector, as is the case with BSW14 and *L. saligna*.

**Part II – Major Issues: Key Experiments Required for Acceptance**

**Reviewer #2: Unfortunately, Western-Blots are required to validate some of the manuscript conclusions, in particular those issued from results presented in Figures 8 and 10.**

We have added Western blots for all the fluorescently-tagged effectors presented in Fig. 5 in lettuce and Fig 6 in *N. benthamiana*. It is not clear what the value of a Western blot is for Fig. 8; all constructs are clearly expressed due their elicitation of reactions in at least two genotypes and all genotypes react to the majority of effectors. For Fig. 10, we have now shown that BSW04m is expressed in Ninja by confocal microscopy and Western blotting (Fig. S3 and S6).

**I am puzzled by the fact that in the first version of the manuscript HopS2 did not suppress the flg22-mediated ROS burst and in this new version it does? How do the authors explain this?**

The first manuscript contained data from three separate dates of assays in which HopS2 was used as a control. However, during re-analysis of the data it was observed that one of the dates had very high variability and the controls did not show strong suppression, so this date was excluded during the analysis presented in this manuscript. The revised manuscript now contains data from three additional assays using fluorescently tagged proteins and HopT1 as the control as suggested by the reviewer.

**Figure 10: I may have missed something, but based on the results presented in the manuscript I don’t understand how the authors narrow down the recognized region to residues 405-458 (there seem to be lots of aa differences C-terminal to this region).**

All of the other C-terminal polymorphisms outside of this region were found to be present in two unrecognized alleles of BSW04m, BSW04m-10C or BSW04m-4C. This is now clarified in the text (page 15).

**Figure S3: I still believe that BLN06 from BL24 should be included in this experiment in order to show that it is recognized by the different lettuce lines in the experimental conditions used by the authors.**

We agree that this would be ideal. However, this is a very minor point and doing this experiment would significantly delay publication. Furthermore, we have deliberately not worked on effectors being studied in other labs.

The interesting result from this experiment—that BLN06 from SF5 causes cell death on NunDm17 but does not have a signal peptide, does not need the BL24 control to remain valid. We have reworded the results paragraph to focus on this result and added a caveat that lack of cell death caused by the SF5 alleles in LS102 would need the BL24 control to be certain that it was not due to a difference in genotype in our stock of LS102 or due to experimental variability.

**“Reviewer #3: Regarding the previous request to provide western data confirming that effector-FP fusion proteins were stably expressed, I agree that this is not required for all 30,000 in planta expressions. And I sympathise with situations where labs have struggled to perform lab work due to the pandemic. It was not a frivolous request for new data it was a request for basic experiments without which conclusions cannot be made. The localisations in Figure 5 may, or may not, be due to full-length effector-FP fusions. They could be due to cleavage products, i.e. the FP is clearly intact but how much of the effector is present? Or, where the localisation is the same as you would expect for FP-only controls (which are missing), how do we know that any effector is present? For me, this Figure adds nothing. For sure, we expect effectors to occupy different subcellular locations, but I can’t conclude anything from the Figure as it stands.”**

The localization results are descriptive but we think they are interesting as part of the characterization of these effectors and may be valuable knowledge for future research into the function of these proteins.

We have now performed and added the requested Western blotting data to the manuscript. One protein, BLR36, was indeed found to be completely cleaved with no full length fusion protein expressed, so we removed it from Figure 5. All other proteins had full length fusions present on the Western blot (Figure S3), with some showing weak smaller bands that may represent cleavage products (BSW11, BSW13, BSW19). However, the localizations for those three effectors are distinct from free GFP (which has been added to Figure 5). Therefore, we believe that the observed cleavage likely occurred during protein extraction rather than *in planta*.

**In Figure 6 the authors indicate that PTI suppression activity of effectors can be altered by the addition of an FP fusion. The ability of the untagged BSW14 and BSW19 to suppress PTI is lost by the FP-fusion versions. In contrast, untagged BSW04p and BSW13 fail to suppress PTI, whereas the tagged versions do. Further, the ability of untagged BSW03 to enhance the ROS burst (an unusual effector activity) is prevented by addition of the FP fusion. Are all of these fusions intact? If not, what do we make of the localisation data in Figure 5?**

As requested, we performed Western blotting on all effector fusions shown in Fig. 6 transiently expressed in *N. benthamiana*. All effectors had bands consistent with the expected size of the fusion protein. Smaller bands which could represent cleavage products, were also observed for all effectors with the exception of BSW04p. These effectors show the same localization in *N. benthamiana* (not shown) as in lettuce (Fig. 5), which is distinct from GFP alone, consistent with cleavage during protein extraction.

**Part III – Minor Issues: Editorial and Data Presentation Modifications**
Please use this section for editorial suggestions as well as relatively minor modifications of existing data that would enhance clarity.

**“Might it be possible to add a supplementary table with the specific IDs of genes in various categories from Fig. 2/TableS2?”**

As requested, we have added a supplementary file (File S2) that contains sequences for the predicted proteins in the various categories from Fig 2/Table S2 from all analyzed oomycete genomes.

**Reviewer #2: Figure 5: I find it difficult to define the subcellular localization for BSW14 and BSW19 based on the provided images.**

Co-expression with organelle markers would be needed to define the subcellular localization of these effectors. We have added a description that they appear to be localized in round bodies to the text.

**“Figure 9: while it is understandable that in Figure 7 only average scores are reported for presentation reasons, here it will be suitable to present also information on dispersion, ideally showing all data as box plots. Also, the Figure refers to 67 RILs but the text states 70 RILS (l. 293). Based on the scores shown for the combination BSW14-ViAE in Figures 7 and 9 there seems to exist a variability that justifies presenting the dispersion.”**

We thank the reviewer for this comment. In Figure 9, we have replaced the scores with bar plots and fixed the text to state 67 RILs.

**“Figure 10: understanding the composition of the different chimeric proteins obtained by domain swaps is difficult; a simple diagram using color-coded boxes will improve the comprehension of the figure. In section C, from the formal point of view it is surprising to see protein domains and aminoacid changes positioned on a nucleotide sequence.”**

As requested, we have updated the figure using color coded boxes and changed sequence to amino acid.

**“Figure S1: the legend is inverted for A and B. Also, it would be nice if the authors explained how the transcript levels are calculated, or gave a reference for the method.”**

The transcript levels were calculated by comparing the cycle thresholds (Ct) of each effector gene and the housekeeping gene in each sample and transforming that number using 2^ΔCt^ to get a relative quantification value. We have added a reference for the method in the supplemental figures and tables file.

**“Discussion, l. 436: if I understand properly, the authors mean BSW04m. Concerning the sentence itself, ideally it should be shown that the protein is expressed in the lettuce line where it does not elicit cell death (there could be differences of expression between lettuce lines).”**

We have deleted this sentence because we have now shown that BSW04m is expressed in Ninja, but not recognized (Figure S6).
